# Association of Myocardial Changes and Gene Expression of the *NFATC1* and *NFATC4*—Calcineurin Signaling Pathway in Children with Bicuspid Aortic Valve

**DOI:** 10.3390/children10091434

**Published:** 2023-08-23

**Authors:** Andrii Kamenshchyk, Margaryta Gonchar, Valentyn Oksenych, Aleksandr Kamyshnyi

**Affiliations:** 1 Department of Hospital Pediatrics, Zaporizhzhya State Medical and Pharmaceutical University, 69035 Zaporizhzhya, Ukraine; andrei.kamens@gmail.com; 2 First Department of Pediatrics and Neonatology, Kharkiv National Medical University, 61000 Kharkiv, Ukraine; margarytagonchar@gmail.com; 3 Broegelmann Research Laboratory, Department of Clinical Science, University of Bergen, 5020 Bergen, Norway; 4 Department of Microbiology, Virology and Immunology, I. Horbachevsky Ternopil State Medical University, 46001 Ternopil, Ukraine; alexkamyshnyi@gmail.com

**Keywords:** bicuspid aortic valve, children, NFATC1 gene expression, myocardial changes, posterior wall thickness, aorta

## Abstract

Background: The role of NFATC gene expression in bicuspid aortic valve (BAV) progression is not fully understood. The aim of this study is to determine the significance of NFATC1 and NFATC4 gene expression for myocardial changes in children with BAV. Methods: In 47 children with BAV, the standard Doppler echocardiographic characteristics were detected, and the expression of the NFATC1 and NFATC4 genes was studied. Results: Posterior wall thickness in diastole (PWTd) and aortic valve peak pressure gradient (AoPPG) in BAV patients were significantly higher compared to healthy controls (PWTd median (min–max), 9 (7–10) mm vs. 7 (6–8) mm; and AoPPG median (min–max), 7.79 (2.98–15.09) mm Hg vs. 2.94 (2.42–3.72) mm Hg). The expression of the NFATC1 gene in BAV children was significantly higher compared to NFATC4 (NFATC1 median (min–max); 70.88 (8.79–106.51) e.u. vs. 7.72 (1.74–22.67) e.u., respectively *p* < 0.05). A significant correlation of NFATC1 expression with Ao found (R = +0.53, *p* < 0.05). In BAV patients with PWTd > 8 mm and Ao > 21 mm the NFATC1 expression was significantly higher compared to those with PWTd ≤ 8 mm and Ao ≤ 21 mm (NFATC1 median (min–max); 45.49 (5.01–101.52) e.u. vs. 15.53 (2.36–44.40) e.u., *p* < 0.05 and 81.11 (20.27–101.10) e.u. mm vs. 12.16 (2.40–45.49) e.u., *p* < 0.05, respectively). Conclusion: In children with BAV the high expression of the NFATC1 calcineurin signaling pathway gene is associated with elevated PWTd and Ao.

## 1. Introduction

Bicuspid aortic valve (BAV) is the most common congenital heart disease with a prevalence ranging from 0.5% to 1.4% in the population and is associated with severe complications, including aortopathy, and may present as asymptomatic in childhood [1,2]. On the other hand, calcineurin signal pathway genes participate in intrauterine valvulogenesis and hypertrophic reactions after birth [3,4]. The role of calcineurin pathway biomarkers in early myocardial changes in children with BAV has been demonstrated in our previous study [5]. Basic genes regulating these reactions belong to the nuclear factor-activated T-cell family (NFATC) with the highest expression in adipocytes, chondrocytes, and during the morphogenesis of veins and heart valves, as well as in myocardial hypertrophy [6,7]. In addition, the recruitment of T cells in myocardial remodeling and heart failure has been demonstrated [8,9]. Homozygous alleles with conservative sequences of the NFATC1 gene were found in 1 out of 4 patients with BAV [10,11]. It has been shown in animal models that overproduce calcineurin is the cause of hypertrophic responses and the development of heart failure, but the overproduction of calcineurin inhibitors may have a protective effect [12,13].

Phenotypes of BAV also demonstrated the heterogeneity in relation to subsequent aortic complications with the prevalence of BAV-RL and BAV with raphe corresponding to the development of aortic regurgitation and BAV-RN corresponding to aortic stenosis but these studies mostly included adolescents and adults [14,15]. These data indicate that aortic complications most commonly develop after pediatric age. It should also be noted that there are different characteristics of BAV in children compared to adults with the predominance of type 0 and type 1 morphology, according to Sievers, and no significant relationships with myocardial remodeling in asymptomatic patients [16]. At the same time, in children with BAV, there are changes in the left ventricle (LV), such as increased LV mass mainly combined with significant aortic stenosis and regurgitation were demonstrated, but aortal dilation was associated with older age [17,18]. 

In line with the above studies and the importance of calcineurin signaling pathway genes in both valve development and myocardial remodeling, these factors could also be implicated in myocardial changes in children with BAV. In this connection, detecting the significance of NFATC1 and NFATC4 expression in early myocardial remodeling in BAV children seems relevant. 

The aim of this study is to determine the association of NFATC1 and NFATC4 gene expression with myocardial remodeling and changes in intracardiac hemodynamics in children with BAV. 

## 2. Materials and Methods

### 2.1. Patient Selection

This study was conducted at the Zaporizhzhya Regional Clinical Children’s Hospital. 112 patients, in whom the diagnosis of BAV was established by general clinical investigation and standard Doppler echocardiography, participated in this study. The control group included 20 healthy children neither pathology of the cardiovascular system nor heart anomalies by their clinical exam and heart ultrasound. The criteria for inclusion in the main study group were the age of patients (from 9 to 15), the diagnosis of BAV, the absence of complaints at presentation, neither ultrasound nor clinical signs of aortopathies or heart complications, and informed consent of children or their parents to conduct this study. 

The exclusion criteria were the age of children younger than 9 and older than 15 years old, participation in any sports, the presence of clinical signs of heart failure, congenital malformations of other organs and systems, established chromosomal abnormalities, and chronic diseases in the stage of decompensation or acute infectious diseases. After inclusion and exclusion criteria were applied, there were 47 patients with BAV in the main group without significant differences in age, BMI or gender distribution to control (Table 1).

### 2.2. Doppler Echocardiography

Doppler echocardiography was performed using the “Xario 100” scanner (Toshiba, Tokyo, Japan) with a 2.5 MHz sensor and detection of standard parameters. The systolic function and ejection fraction of the LV were assessed by M-mode echocardiography. The parameters of the hemodynamics at the valves were assessed by Doppler measurements. Relative left ventricular posterior wall thickness (RPWT) was detected by A. Ganau [19]. Left ventricular mass (LVM) detected by Devereux R.B [20]. Left ventricular myocardium mass index (LVMMI) was detected by P. Gosse [21]. 

### 2.3. Genetic Study

The expression level of NFATC1 and NFATC4 genes in the blood cells of patients was detected by total RNA extraction, and the obtaining of cDNA was achieved by reverse transcription using a CFX96™ Real-Time PCR Detection Systems amplifier (Bio-Rad Laboratories, Inc., Hercules, CA, USA) and a Maxima SYBR Green/ROX qPCR Master Mix (2X) reagent kit (Thermo Scientific, Waltham, MA, USA). Statistical analysis of PCR data was performed using CFX Manager ™ v3.0 software (Bio-Rad, Hercules, CA, USA). The obtained values were presented in units of relative normalized expression compared to the expression in the control group and were taken as the 1 unit of expression (e.u.). In 47 children with BAV, the expression of the NFATC1 and NFATC4 genes was detected at the basal level and in 3 subgroups divided according to the posterior wall thickness in diastole of more than 8 mm, the aortic root diameter of more than 21 mm and the peak pressure gradient at the aortic valve of more than 6 mm Hg.

### 2.4. Statistical Analysis

Statistical analysis was performed using the Statiatica 13.0 program. The normality of distribution was assessed using the Shapiro–Wilk test. The data were presented in the form M ± m, where M is the sample mean, and m is the error of mean in the case of a normal distribution and in the form of median (min–max) in the case when the distribution deviated from normal. In assessing the significance between groups with normal distribution, the Student’s *t*-test was scored for the presence of the difference between groups, and in the non-normal distribution the Mann–Whitney nonparametric criterion for small samples was scored. The relationship between parameters was assessed by Spearman correlations and presented in the form of a correlation matrix. In order to evaluate the multiple correlations between Doppler echocardiographic parameters and the expression of the studied NFATC genes, the correlation matrix was transformed into a gradient color pattern, with the color changing from white to deep blue for high positive R coefficients and from white to deep red for high negative R values, with numbers ranging from +1.0 to −1.0, respectively. Significant correlation coefficients were marked on the gradient pattern. 

### 2.5. Ethics 

All procedures performed in studies involving human participants were in accordance with the ethical standards according to the principles of the Declaration of Helsinki, and this study was approved by Zaporizhzhya State Medical University Clinical Research Ethical Committee (Protocol Number 5 from 17 April 2019). 

## 3. Results

In children with BAV, significant differences in Doppler echocardiographic parameters were observed between groups (Table 2). 

These included increased Ao, SWTd and PWTd, AoPPG, LVDD, LVM, LVMMI, RPWT and the acceleration of the AVV (*p* < 0.05), which clearly reflected the morphological and functional characteristics of this defect. At the same time, there were no significant differences in other Doppler echocardiographic data between the groups. Notably, RPWT and LVMMI were significantly higher in 29 (61.7%) of the children with BAV with PWTd > 8 mm compared to those with PWTd < 8 mm (*p* < 0.05), which was also associated with a significantly increased Ao, as shown in Table 3. Thus, in two-thirds of the patients, LV enlargement is according to PWTd, LVMMI, and RPWT parameters, which correspond to the concentric hypertrophy of the LV myocardium. In the other 18 patients with BAV (38.3%), LV geometry remained unchanged.

The evaluation of NFATC1 and NFATC4 gene expression in children with BAV has revealed a significantly higher expression of the NFATC1 gene (NFATC1 median (min-max) 70.88 (8.79–106.51) e.u. vs. 7.72 (1.74–22.67) e.u., respectively *p* < 0.05), although the expression of both genes was 71-fold and 8-fold higher compared to control, respectively.

**Figure 1:**
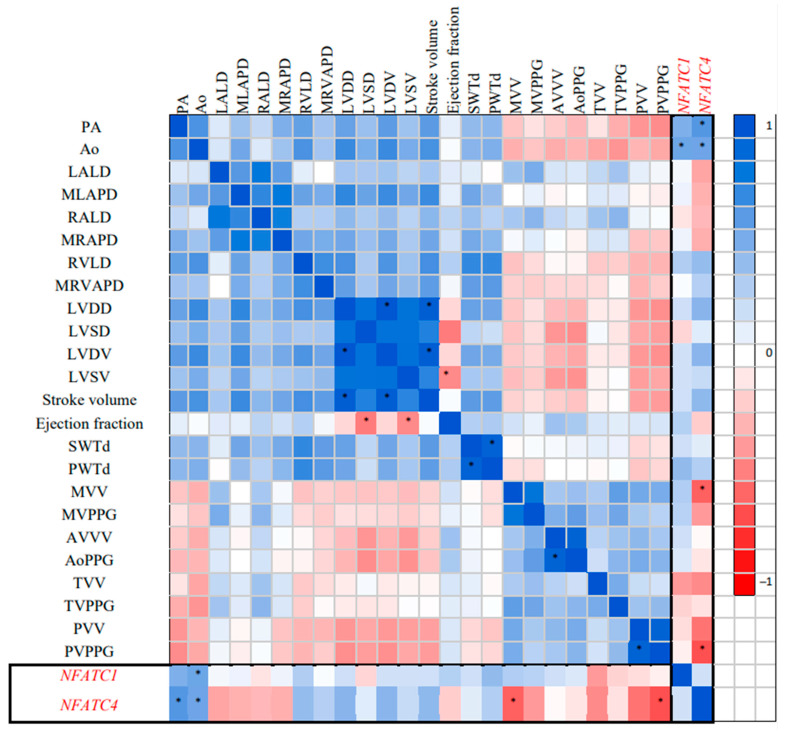
Gradient pattern of correlation matrix for NFATC1 and NFATC4 expression with Doppler echocardiographic parameters in children with BAV (*n* = 47). * Correlations marked at *p* < 0.05.

**Figure 2:**
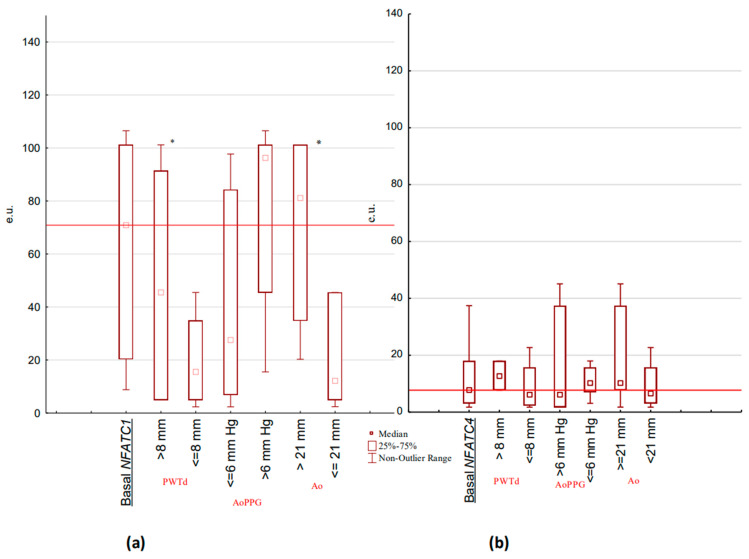
NFATC1 and NFATC4 gene expression levels associated with PWTd, AoPPG and Ao values in children with BAV: (**a**) NFATC1 basal expression (*n* = 47) in PWTd > 8 mm (*n* = 29), AoPPG > 6 mm Hg (*n* = 15), and Ao > 21 mm (*n* = 19); (**b**) NFATC4 basal expression (*n* = 47) in PWTd > 8 mm (*n* = 29), AoPPG > 6 mm Hg (*n* = 15), and Ao > 21 mm (*n* = 19). Basal *NFATC1*–level of *NFATC1* gene basal expression. Basal *NFATC4*—level of *NFATC4* gene basal expression. e.u.—units of relative normalized expression. PWTd—posterior wall thickness in diastole. Ao—aortic root diameter. AoPPG—aortic valve peak pressure gradient. * *p* < 0.05.

The correlation analysis of NFATC1 and NFATC4 was performed using the Doppler echocardiographic parameters (Figure 1). A significant correlation of NFATC1 and NFATC4 with Ao (R = +0.53 and R = +0.54, respectively, *p* < 0.05), and NFATC4 with MVV and PVPPG was shown (R = −0.62 and R = −0.68, respectively, *p* < 0.05). Other significant correlations reflected interactions in functional Doppler echocardiographic parameters of BAV children (LVDD with LVSD and LVDV (R = +0.84 and R = +0.98, respectively, *p* < 0.05); stroke volume with LVDD and LVSD (R = +0.95 and R = +0.74, respectively, *p* < 0.05); ejection fraction with LVSD and LVSV (R = −0.52 and R = −0.46, respectively, *p* < 0.05); MLAPD to LALD (R = +0.61, *p* < 0.05)). The expression levels of the NFATC1 and NFATC4 genes in the change of defect-associated parameters of Doppler echocardiography in children with BAV (Figure 2) showed significant differences.

In children with an increased PWTd of more than 8 mm compared to BAV children with PWTd of less than or equal to 8 mm, the level of NFATC1 expression was significantly higher (NFATC1 median (min–max); 45.49 (5.01–101.52) e.u. vs. 15.53 (2.36–44.40) e.u., *p* < 0.05) with respect to the insignificant differences in NFATC4 caused by this parameter (NFATC4 median (min–max); 12.60 (7.72–37.41) e.u. vs. 6.31(1.74–45.09) e.u., *p* > 0.05). For both NFATC1 and NFATC4 expression in AoPPG of more than 6 mm Hg compared to value less than or equal to 6 mm Hg, there was no significance detected (NFATC1 median (min-max), 96.25 (15.50–106.50) e.u. vs. 27.53 (2.36–91.54) e.u.), *p* > 0.05; and NFATC4 median (min-max), 6.24 (1.74–45.09) e.u. vs. 10.16 (3.08–17.96) e.u., *p* > 0.05). The parameter of Ao related to the value of 21 mm was found to be significantly increased for NFATC1 expression (NFATC1 median (min–max); 81.11 (20.27–101.10) e.u. at Ao >= 21 mm vs. 12.16 (2.40–45.49) e.u. at Ao < 21 mm *p* < 0.05) and was not significant for NFATC4 expression (NFATC4 median (min-max); 10.16 (1.45–45.09) e.u. at Ao >= 21 vs. 6.38 (1.74–22.67) e.u. at Ao < 21 mm *p* > 0.05).

## 4. Discussion

The genes of the NFATC family as transcription factors play an essential role in many pathological processes, such as myocardial hypertrophy tumorigenesis and immune response. The study of the expression of these genes in children with BAV has both diagnostic and prognostic value because of involvement in valvulogenesis and the presence of polymorphisms that could influence the development of this anomaly. Our previous investigation showed the predominance of the rs11665469 homozygous TT genotype of NFATC1 in children with BAV [22], and in the current study, we found an increased expression activity of this gene. It was also found that there was a significant correlation between the expression of NFATC1 and the diameter of the aortic root in children with BAV and its absence of PWTd, although the expression level was significantly increased at elevated values of both parameters. This may indicate an earlier involvement of aortic changes in the pathological process influenced by NFATC1 expression. This is indirectly confirmed by the highest levels of expression of NFATC1 in relation to the other parameters studied and by exceeding the overall level of expression of this gene in children with BAV. Nevertheless, the elevated expression of NFATC1 could also be related to an advanced adaptive change of the LV marked in the BAV patients. Our results are consistent with studies showing that high levels of transcriptionally active mutated gene expression are involved in heart development [23] and may also contribute to autonomous LV remodeling in some children with BAV. 

The established association of NFATC1 gene expression levels with LV and aortic changes in children with BAV allow us to define a risk stratification strategy regarding complications and future cardiovascular events in these patients who are experiencing increasing aortic diameter. High NFATC1 expression in association with changes in PWTd and Ao in children with BAV is indicative of intense and progressive myocardial changes and requires closer follow-ups with clinical evaluation and Doppler echocardiography.

### Limitations

This study has some limitations due to overlap in the parameters studied. Some patients appeared to have both elevated PWTd and Ao, or Ao with elevated AoPPG. Another limitation concerns some phenotypic heterogenicity in BAV anatomy that could affect AV hemodynamics and was not included in this study, However, the association of LV and aortic changes with the level of NFATC1 expression, but not with AV hemodynamics, was found to reflect a predominant remodeling caused by a possible increased expression of mutant genes of the transcriptional cascade associated with BAV. In addition, the results obtained from the expression of NFATC1 in children with BAV could be the basis for a therapeutic approach based on expressive activity regulation.

## 5. Conclusions

The calcineurin signaling pathway gene NFATC1, but not NFATC4, is highly expressed in children with BAV. This increased expression of NFATC1 in children with BAV is associated with myocardial changes in elevated PWTd and also increased Ao. The expression of this gene could indicate possible cardiovascular complications in these patients.

## Figures and Tables

**Table 1:** Demographical information about patients.

Parameters, Units	Children with BAV (*n* = 47)		Control Group (*n* = 20)	
	M ± m		M ± m	
Age (years old)	10.83 ± 0.66		11.00 ± 0.70	
BMI (kg/m^2^)	17.09 ± 0.70		17.93 ± 0.83	
Gender distribution, *n*, (%)	males	females	males	females
	35 (75)	12 (26)	13 (65)	7 (35)

**Table 2:** Doppler echocardiographic characteristics in children with BAV.

Parameter, Units	Children, BAV (*n* = 47)	Control (*n* = 20)	*p*
PA (mm)	20.00 (18.0–20.00)	20.00 (17.0–22.00)	0.48
Ao (mm)	21.38 ± 0.76	19.25 ± 0.45	0.02
LALD (mm)	25.00 (22.00–30.00)	23.0 (18.00–25.00)	0.09
MLAPD (mm)	24.00 (21.00–28.00)	24.00 (20.00–25.00)	0.14
RALD (mm)	25.00 (22.00–30.00)	24.00 (19.00–26.00)	0.13
MRAPD (mm)	24.00 (21.0–27.00)	23.0 (19.0–27.00)	0.11
RVLD (mm)	45.00 (39.00–48.00)	42.50 (40.00–50.00)	0.51
MRVAPD (mm)	22.00 (21.00–23.50)	21.50 (21.00–24.00)	0.11
LVDD (mm)	39.90 (17.60–58.90)	21.50 (14.00–31.00)	0.001
LVSD (mm)	24.80 (21.05–28.40)	28.15 (23.00–31.80)	0.65
SWTd (mm)	8.00 (7.00–11.00)	7.00 (6.00–8.00)	0.04
PWTd (mm)	9.00 (7.00–10.00)	7.00 (6.00–8.00)	0.004
LVM (g)	121.41 ± 13.18	85.90 ± 6.35	0.03
LVMMI (g/m^2.7^)	42.80 ± 3.99 (16.43–54.30)	32.21 ± 2.28 (14.94–29.10)	0.02
RPWT (mm)	0.49 ± 0.04	0.35 ± 0.01	0.01
LVDV (mL)	68.00 (50.00–96.00)	77.00 (60.50–103.50)	0.49
LVSV (mL))	21.50 (15.5–30.50)	25.00 (17.00–38.00)	0.87
Stroke volume (mL)	53.50 (45.50–88.50)	47.00 (38.00–66.00)	0.57
Ejection fraction (%)	69.00 (65.00–72.00)	67.00 (63.00–71.00)	0.07
MVV (m/s)	0.90 (0.82–1.03)	0.82 (0.77–0.99)	0.22
MVPPG (mm Hg)	1.52 (1.02–3.66)	1.22 (1.04–1.85)	0.70
AVV (m/s)	1.66 (0.82–1.99)	0.86 (0.81–1.16)	0.005
AoPPG (mm Hg)	7.79 (2.98–15.09)	2.94 (2.42–3.72)	0.002
TVV (m/s)	0.61 (0.55–0.74)	0.58 (0.55–0.70)	0.98
TVPPG (mm Hg)	0.90 (0.78–1.30)	0.89 (0.78–1.59)	0.83
PAV (m/s)	0.90 (0.85–0.97)	0.90 (0.88–1.06)	0.57
PVPPG (mm Hg)	3.22 (2.98–4.10)	3.57 (3.04–4.50)	0.75

PA—Diameter of pulmonary artery. Ao—aortic root diameter. LALD—left atrial longitudinal dimension. MLAPD—maximum left atrial anterior-posterior dimension. RALD—right atrial longitudinal dimension. MRAPD—maximum right atrium posterior anterior dimension. MRVAPD—maximum right ventricle anterior–posterior dimension. RVLD—right ventricle longitudinal dimension. LVDD—left ventricular end diastolic dimension. LVSD—left ventricular end systolic dimension. SWTd—septal wall thickness in diastole. PWTd—posterior wall thickness in diastole. LVM—left ventricular mass. LVMMI—left ventricular myocardium mass index. RPWT—relative left ventricular posterior wall thickness. LVDV—left ventricular end diastolic volume. LVSV—left ventricular end systolic volume. MVV—mitral valve bloodstream velocity. MVPPG—mitral valve peak pressure gradient. AVV—aortic valve bloodstream velocity. AoPPG—aortic valve peak pressure gradient. TVV—tricuspid valve bloodstream velocity. TVPPG—tricuspid valve peak pressure gradient (mm Hg). PAV—pulmonary artery valve bloodstream velocity. PVPPG—pulmonary artery valve peak pressure gradient.

**Table 3:** Parameters of the left ventricle in dependence of PWTd in children with BAV.

Parameters, Units	Children with BAV PWTd > 8 mm (*n* = 29)	Children with BAV PWTd ≤ 8 mm (*n* = 18)	*p*
RPWT (mm)	0.49 ± 0.04	0.39 ± 0.02	0.02
LVMMI (g/m^2.7^)	60.10 (32.14–105.75)	32.59 (18.85–56.37)	0.003
Ao (mm)	24.16 ± 0.73	20.25 ± 1.23	0.02
AVV (m/s)	1.3 (0.63–3.6)	1.1 (0.75–2.8)	0.68
AoPPG (mm Hg)	4.71 (1.87–8.09)	3.98 (2.09–8.60)	0.23

RPWT—Relative left ventricular posterior wall thickness. LVMMI—left ventricular myocardium mass index. Ao—aortic root diameter. AVV—aortic valve bloodstream velocity. AoPPG—aortic valve peak pressure gradient.

## Data Availability

Most of the data are included in the current manuscript, except the data covered by privacy restrictions.
